# Implementation of pneumatic retinopexy in the Japanese population

**DOI:** 10.1007/s10384-025-01241-z

**Published:** 2025-07-25

**Authors:** Kunihiko Akiyama, Takaaki Matsuki, Ken Watanabe, Aurora Pecaku, Sumana Naidu, Rajeev H. Muni

**Affiliations:** 1 Department of Ophthalmology, NHO Tokyo Medical Center, 2-5-1, Higashigaoka, Meguro-ku, Tokyo, 152-8902 Japan; 2 Division of Vision Research, National Institute of Sensory Organs, NHO Tokyo Medical Center, Tokyo, Japan; 3 Department of Ophthalmology and Vision Science, University of Toronto, Toronto, ON Canada; 4 Department of Ophthalmology, St. Michael’s Hospital, Unity Health Toronto, Toronto, ON Canada

**Keywords:** Implementation, Social media, Pneumatic retinopexy, Rhegmatogenous retinal detachment, Reattachment rate

## Abstract

**Purpose:**

To propose an implementation model for pneumatic retinopexy (PnR) in a region where PnR is performed infrequently, and to assess its impact on treatment of rhegmatogenous retinal detachment (RRD).

**Study design:**

Retrospective case series.

**Methods:**

We reviewed 222 consecutive eyes with primary RRD treated from July 2017 to September 2023 at a tertiary care center in Japan. The treatment methods utilized included pars plana vitrectomy (PPV), scleral buckling (SB) and PnR. The surgeon learned PnR through social media. Primary anatomic reattachment rate (PARR) and visual acuity outcomes were compared between the pre-PnR (prior to the implementation; 110 eyes) and post-PnR (after the implementation; 112 eyes) periods, as well as between PnR and PPV in the post-PnR period. PARR for PnR was also evaluated based on RRD characteristics and gas injection frequency.

**Results:**

In the post-PnR period PnR was performed in 53.6% (60/112)of cases. The PARR was similar in the pre-PnR (97.3%) and post-PnR (93.8%) periods (*P*=.33). Visual outcomes were similar both across periods and between PnR and PPV at 3, 6 and 12 month post-operatively. The PARR for PnR was 88.3% overall, 90.5% in eyes meeting the Primary Rhegmatogenous Retinal Detachment Outcomes Randomized Trial (PIVOT) criteria, 93.3% in eyes with a single break and 100% in eyes with a single break meeting PIVOT criteria. Eyes with a single gas injection had higher PARR than eyes requiring an additional gas injection (93.5% vs. 71.4%).

**Conclusion:**

Remote-learning utilizing social media effectively enabled PnR implementation with favorable anatomic and functional outcomes in a real-world setting in Japan.

**Supplementary Information:**

The online version contains supplementary material available at 10.1007/s10384-025-01241-z.

## Introduction

Pneumatic retinopexy (PnR) is a minimally invasive, office-based procedure for rhegmatogenous retinal detachment (RRD) repair which involves intravitreal injection of expansile gas and retinopexy with laser or cryopexy [1]. Randomized clinical trials validate this procedure in terms of post-treatment visual acuities being superior to those following scleral buckling (SB) surgery [2, 3] and pars plana vitrectomy (PPV) [4]. The Pneumatic Retinopexy versus Vitrectomy for the Management of Primary Rhegmatogenous Retinal Detachment Outcomes Randomized Trial (PIVOT) demonstrates an 80.8% primary reattachment rate in patients who underwent PnR and met the inclusion criteria of the trial [4]. Advantages of PnR are reported in comparison with PPV, such as less vertical metamorphopsia, superior vision-related quality of life in the first 6 months, superior ability to restore photoreceptor integrity and less outer retinal folds and retinal displacement [4–10].

Despite such versatility and efficacy, the use of PnR as a primary procedure for RRD repair varies widely among clinical practices and geographic regions. A nationwide database examining trends in vitreoretinal surgery in Japan only analyzed trends for patients undergoing PPV or SB for RRD, implying a low uptake of PnR in Japan [11–13], although a similar procedure had been adopted in 1984 by Emi et al. [14] and was utilized widely in Japan during the 1980’s and 1990’s [15–19]. Similarly, a national database study in Korea describing trends in surgical approaches for RRD excluded patients treated with PnR [20].

Several reasons are assumed for the reduced uptake of PnR among some surgeons. In some areas or countries, easy access to operating rooms and sufficient number of vitreoretinal surgeons may lead patients as well as surgeons to select PPV or SB rather than PnR. Another reason could be the historical emphasis on the primary anatomic reattachment rate (PARR) which is usually better with PPV or SB. However, other outcomes such as visual acuity, vertical metamorphopsia, and anatomic outcomes of integrity have been shown to be superior with PnR vs PPV [4, 5]. These recent data may compel some surgeons to choose the repair procedure based on the outcome that is most important to a given patient. Additionally, surgeons may lack opportunities for training and experience with this procedure since many fellowships do not teach PnR [21]. Without instructions or experience, it could be challenging to overcome difficult situations during PnR procedures. Nonetheless, an efficacious solution emerged amidst the surge in digital learning during the COVID-19 pandemic [22]. This technology provides a user-friendly, readily accessible, and cost-effective means to enhance collaborative learning and foster surgical innovation [22, 23]. The use of social media to learn surgical techniques results in efficient and effective sharing of information and knowledge of novel procedures [24–27].

Here, we propose an implementation model of PnR in a tertiary care center in Japan utilizing social media and aim to assess the implementation and outcomes of PnR and the distribution of procedures performed to treat RRD before and after the implementation of PnR.

## Materials and methods

### Study design

This was a retrospective cohort study of patients with RRD who presented to a tertiary vitreoretinal surgery practice at the National Hospital Organization Tokyo Medical Center, Tokyo, Japan from July 2017 to September 2023. Ethics Committee approval was obtained (approved number: R24-037), all participants provided informed consent for the treatment procedure, and the study was carried out following the Declaration of Helsinki.

### Patients and observations

Inclusion criteria were patients who were treated for RRDs with PnR, SB, or PPV, and had a minimum of 3-month postoperative follow-up. RRDs complicated with proliferative vitreoretinopathy (PVR) grade C and coexisting exudative or tractional detachment were excluded. The treatment method was selected at the surgeon’s discretion, but generally, eyes that were judged eligible for PnR were treated by this method, particularly when they had met PIVOT criteria illustrated as “a single retinal break or a group of breaks in detached retina within 1 clock hour above the 8- and 4-o'clock meridians, with any number, location and size of retinal breaks or lattice degeneration in attached retina [4].” All patients included in this study were treated by a single surgeon (KA).

The procedures for PnR were similar to those presented and standardized in the PIVOT trial [4]. Briefly, the anterior chamber was tapped, and 100% sulfur hexafluoride was injected into the vitreous cavity with the minimum volume of 0.6 ml and 0.3 ml in excess of the volume of the anterior chamber tap. Photocoagulation was applied using slit lamp equipment to the causative retinal breaks as soon as they were reattached, usually on the day after the gas injection. In some cases, cryopexy was performed when gas was injected instead of or in addition to laser photocoagulation. Any retinal breaks or lattice degeneration in the attached retina were treated with laser retinopexy prior to gas injection. Additional gas injection was performed/pre-planned when needed or thought to be advantageous [28] and reattachment after sequential injections was considered as a primary anatomical reattachment [4, 29–31] whenever the duration between injections was two weeks or shorter.

Data were collected from the electronic medical records. The following variables were extracted: demographic information, clinical characteristics (refraction, lens status, foveal status, RRD extent, location, number and type of breaks, presence of a giant retinal tear, presence of vitreous hemorrhage and duration of the symptoms), surgical interventions (PnR, PPV or SB), number of gas injections for PnR, and postoperative outcomes including anatomic reattachment at 3-months post-operatively and best corrected visual acuity (BCVA) at baseline, three, six and twelve months post-operatively. Details regarding PnR failures were also collected.

### Primary outcomes and measures

The primary outcomes were the difference in the primary anatomic reattachment rate (PARR) and visual acuity between the two periods (before and after implementation of PnR), and between eyes treated with PnR and PPV. Periods were defined based on the time when the surgeon implemented pneumatic retinopexy (PnR) in his practice (December 2020) and were classified as the pre-PnR period versus the post-PnR period. For comparison of visual acuity outcomes between procedures, patients in the post-PnR group were further divided into two sub-groups: patients treated with PnR and with PPV (patients treated with SB were excluded from these sub-groups because of their small number). Demographic information, clinical characteristics, PARR and BCVA at each time point were compared between the two periods (pre- vs. post-PnR), and between the sub-groups (PnR vs. PPV). The visual acuity was tested using a Landolt chart and was converted into the logarithm of the minimum angle of the resolution (logMAR) units for statistical analyses. For the assessment of visual acuities, eyes with previous history of macular disorder, amblyopia or dense vitreous hemorrhage at baseline were excluded.

### Statistical analyses

All statistical analyses were performed using the statistical software IBM SPSS version 26 (IBM Corp., 2019). The Chi-square test and Fisher exact test were used to assess the categorical variables, and the 2-sided independent sample *t-*test or Mann-Whitney test was used to assess the continuous variables. Quade non-parametric ANCOVA test was used for assessment of visual outcomes controlling for the baseline foveal status and the symptom duration > 30 days. Continuous variables are presented as mean with standard deviation (SD) or median with IQR if normal distribution was not confirmed, and categorical variables are presented as absolute values and percentages. Results were considered statistically significant if the *P*-value was less than 0.05. The sample size required to confirm non-inferiority in PARR between the pre-PnR and post-PnR groups was estimated as 248 cases (non-inferiority margin set at 10%) and 4642 cases (non-inferiority margin set at 5%) with 80% statistical power.

## Results

Two hundred twenty-two patients were included. Of these, 110 eyes (49.5%) and 112 eyes (50.5%) belonged to the pre-PnR and post-PnR periods, respectively.

The demographic and baseline characteristics are presented in Table 1. Patients in both the pre-PnR and post-PnR periods were similar. Three cases in the post-PnR period had previous history of macular disorders or amblyopia (macular dystrophy, macular degeneration and amblyopia). The follow-up period was significantly longer in the pre-PnR period, although this was not thought to impact the results as we compared PARR and the visual acuity outcomes at the specific time points.

**Table 1:** Demographics, baseline characteristics and follow-up time

Characteristics	Total cases (n=222) No. (%)/Average± SD	Pre-PnR period (n=110) No. (%)/Average± SD	Post-PnR period (n=112) No. (%)/Average± SD	*P*
Sex				0.81^a^
Male Female	137 (61.7) 85 (38.3)	67 (60.9) 43 (39.1)	70 (62.5) 42 (37.5)	
Age (years)	52.7 ± 14.4	52.3 ± 12.4	53.0 ±16.3	0.74^b^
Eye				0.51^a^
Right Left	116 (52.3) 106 (47.7)	55 (50.0) 55 (50.0)	61 (54.5) 51 (45.5)	
Refraction (diopters)	-4.35 ± 3.96	-4.25 ± 3.73	-4.45 ±4.20	0.94^c^
Lens status				0.09^a^
Phakia Pseudophakia	173 (77.9) 49 (22.1)	91 (82.7) 19 (17.3)	82 (73.2) 30 (26.8)	
Foveal status				0.38^a^
On Off Splitting	118 (53.2) 79 (35.6) 25 (11.2)	54 (49.1) 41 (37.3) 15 (13.6)	64 (57.2) 38 (33.9) 10 (8.9)	
Quadrants of RRD				0.21^d^
1 2 3 4	61 (27.5) 115 (51.8) 38 (17.1) 8 (3.6)	24 (21.8) 61 (55.5) 22 (20.0) 3 (2.7)	37 (33.0) 54 (48.2) 16 (14.3) 5 (4.5)	
Location of breaks				0.68^d^
Superior Inferior Superior+Inferior Temporal/Nasal Macular hole Undetected	133 (59.9) 33 (14.9) 29 (13.1) 22 (9.9) 2 (0.9) 3 (1.4)	68 (61.8) 14 (12.7) 16 (14.5) 10 (9.1) 0 (0.0) 2 (1.8)	65 (58.0) 19 (17.0) 13 (11.6) 12 (10.7) 2 (1.8) 1 (0.9)	
Number of breaks	1.9 ± 1.2	1.9 ± 1.3	1.9 ± 1.1	0.48^c^
Type of breaks				0.89^d^
Flap Lattice edge Hole in lattice Atrophic Dialysis Others	85 (38.8) 68 (31.1) 32 (14.6) 15 (6.8) 13 (5.9) 6 (2.7)	41 (37.6) 36 (33.0) 17 (15.6) 6 (5.5) 7 (6.4) 2 (1.8)	44 (40.0) 32 (29.1) 15 (13.6) 9 (8.2) 6 (5.5) 4 (3.6)	
Giant break	12 (5.4)	5 (4.5)	7 (6.3)	0.57^d^
Vitreous hemorrhage				0.65^d^
No Slight Dense	169 (76.1) 46 (20.7) 7 (3.2)	87 (79.1) 20 (18.2) 3 (2.7)	82 (73.2) 26 (23.2) 4 (3.6)	
Duration > 30 days^e^	40 (18.7)	19 (17.6)	21 (19.8)	0.68^a^
Follow-up (months)	24.5 ± 20.2	37.6 ± 20.8	11.6 ± 6.9	<0.001^c^

*PnR* pneumatic retinopexy, *RRD* rhegmatogenous retinal detachment

^a^Chi-square test

^b^Independent samples *t*-test

^c^Mann-Whitney test

^d^Fisher’s exact test

^e^Cases with unknown duration (n=8) are excluded

Although the lens status was similar at baseline in the two groups (Table 1), it was significantly different after RRD repair between the subgroups (PnR and PPV procedures) because the majority of patients treated with PPV underwent combined cataract surgery, which is a common practice in Japan [11, 32]. Consequently, 71.7% (43/60) of PnR cases were phakic and 97.1% (34/35) of PPV cases were pseudophakic during the follow-up period (Table 2). The postoperative lens status among cases treated for PPV did not differ between the two periods (*P*=0.67).

**Table 2:** Post-treatment lens status in the post-pneumatic retinopexy period

Lens status	Pneumatic retinopexy (n=60) No. (%)	Pars plana vitrectomy (n=35) No. (%)	*P* Value
Phakia Pseudophakia	43 (71.7) 17 (28.3)	1 (2.9) 34 (97.1)	<0.001*

*Chi-square test

Distribution of the procedures is demonstrated in Table 4. In the pre-PnR period, PPV was the most common procedure. With PnR implementation, the distribution of surgical procedures favored PnR as the most common, performed in 53.6% (60/112) of all procedures. When examining PPV and SB procedures only, the distribution among the two procedures was similar between the two periods (*P*=0.18).

### Primary and secondary reattachment rate and complications

The PARR was 97.3% (107/110) and 93.8% (105/112) for the pre- and post-PnR periods, respectively, with no statistically significant difference (*P*=0.33) (Table 5). The PARR in patients who underwent PnR in the post-PnR period was 88.3% (53/60). Among the 42 patients meeting the criteria outlined in the PIVOT trial [4], a PARR of 90.5% (38/42) was achieved, whereas it was 83.3% (15/18) for those not meeting the PIVOT criteria (Table 5). Additional gas injection was performed in 14 patients, and the PARR was lower among them compared to PnR cases treated with a single injection (71.4% vs. 93.5%) (Table 6). Of these 14 patients, a single retinal break ≥ 1 clock hour, multiple breaks ≥ 1 clock hour and inferior multiple breaks were found in 4, 3 and 1 patients, respectively. Additional gas was injected in two cases meeting PIVOT criteria because the size of the bubble was much smaller than expected after injection. PARR in eyes with a single retinal break (regardless of the size and distribution of the breaks) was even higher than in eyes meeting the PIVOT criteria (93.3% vs. 90.5%), and was 100% (25/25) in eyes with a single break meeting the PIVOT criteria (Table 6). The secondary anatomic reattachment rate was 100% in both groups.

Recurrence of RRD was documented in one case after SB in the post-PnR period. This case was considered as a primary reattachment because recurrence occurred more than one year after the initial surgery. One case in the post-PnR period was complicated with vitreous hemorrhage after SB surgery and was treated with subsequent PPV. None of the eyes with primary reattachment after PnR experienced any additional surgery due to recurrence of RRD or post-treatment complications such as cataract and epiretinal membrane during the follow-up period. The very low redetachment rate after PnR in this study is consistent with the results recently published in a post-hoc analysis of the PIVOT trial that reported very low and similar long-term redetachment rates following PnR vs. PPV [33].

### Visual outcomes

After controlling for the foveal status and duration of symptoms, visual acuities were similar between the pre- and post-PnR periods and between PnR and PPV at every timepoint (Table 3), indicating that implementation of PnR did not have any negative impact on the visual acuity outcomes.

**Table 3:** Comparison of visual acuities (excluding cases with previous macular disorder, amblyopia, or dense vitreous hemorrhage)

Visual acuity (LogMAR)	Pre-PnR period (n=107) Median (IQR)	Post-PnR period (n=105) Median (IQR)	*P*^a^
Baseline	0.10 (-0.08 to 0.52)	0.05 (-0.08 to 0.40)	0.98
3 months	0.00 (-0.08 to 0.10) (n=107)	0.00 (-0.08 to 0.16) (n=102)	0.99
6 months	-0.08 (-0.08 to 0.06) (n=98)	-0.08 (-0.08 to 0.05) (n=89)	0.91
12 months	-0.08 (-0.08 to 0.00) (n=89)	-0.08 (-0.08 to 0.10) (n=59)	0.24

Visual acuity (LogMAR)	Pneumatic retinopexy (n=58) Median (IQR)	Pars plana vitrectomy (n=31) Median (IQR)	*P*^b^
Baseline	0.00 (-0.08 to 0.33)	0.22 (0.00 to 0.70)	0.12
3 months	-0.08 (-0.08 to 0.10) (n=58)	0.00 (-0.08 to 0.22) (n=30)	0.49
6 months	-0.08 (-0.08 to 0.01) (n=54)	0.00 (-0.08 to 0.30) (n=26)	0.64
12 months	-0.08 (-0.08 to 0.00) (n=34)	-0.02 (-0.08 to 0.22) (n=20)	0.55

There were some missing data as indicated by the number of patients at each time point, because some patients skipped some follow-up visits, and some were lost to follow-up during the study period

*PnR* pneumatic retinopexy

^a^Compared between the two periods controlling for baseline foveal status and duration>30 days (Quade non-parametric ANCOVA)

^b^Compared between the two procedures in the post-PnR period, controlling for baseline fovea status and duration>30 days (Quade non-parametric ANCOVA). Eyes treated with scleral buckling are not included

### Failure after pneumatic retinopexy

Seven eyes underwent PPV due to failure after PnR. Retinal reattachment was achieved with a single additional surgery in all these cases. Details of these cases are provided in the Online Resource. There was a preponderance of male gender (6 cases) and retinal breaks associated with lattice degeneration (4 cases) among the failed PnR cases. Five of the 7 eyes were myopic with a refraction of less than -3.0 diopters. None of the failures were related to any adverse event associated with the PnR procedures except for a case with subretinal migration of gas through a relatively large perivascular break.

## Discussion

In this study, we aimed to evaluate the implementation of PnR in a tertiary care center in Japan where PnR had almost never been performed at the time of implementation. The baseline characteristics were well-balanced between the two periods (Table 1). The outcomes and distribution of PPV and SB procedures were similar between the two periods (Table 4 and 5).

**Table 4:** Distribution of procedures

Procedure	Total cases (n=222) No. (%)	Pre-PnR period (n=110) No. (%)	Post-PnR period (n=112) No. (%)	*P*
Pneumatic retinopexy	60 (27.0)	0 (0.0)	60 (53.6)	NA
Scleral buckling	42 (18.9)	25 (22.7)	17 (15.2)	NA
Pars plana vitrectomy	120 (54.1)	85 (77.3)	35 (31.2)	0.18*

*PnR* pneumatic retinopexy

*Comparison of distribution of scleral buckling and pars plana vitrectomy between the two periods by chi-square test

**Table 5:** Primary anatomic reattachment rate

Procedures	Pre-PnR period (n=110) No. (%)	Post-PnR period (n=112) No. (%)	*P*^a^
Overall (n=222)	107 (97.3%)	105 (93.8%)	0.33
Pneumatic retinopexy (n=60) Meeting PIVOT criteria (n=42) Not meeting PIVOT criteria (n=18)	NA NA NA	53/60 (88.3%) 38/42 (90.5%) 15/18 (83.3%)	0.42^b^
Scleral buckling (n=42)	24/25 (96.0)	17/17 (100.0%)	1.00
Pars plana vitrectomy (n=120)	83/85 (97.6)	35/35 (100.0%)	1.00

*PnR* pneumatic retinopexy

^a^Fisher’s exact test

^b^Comparison between cases meeting and not-meeting PIVOT criteria

**Fig. 1:**
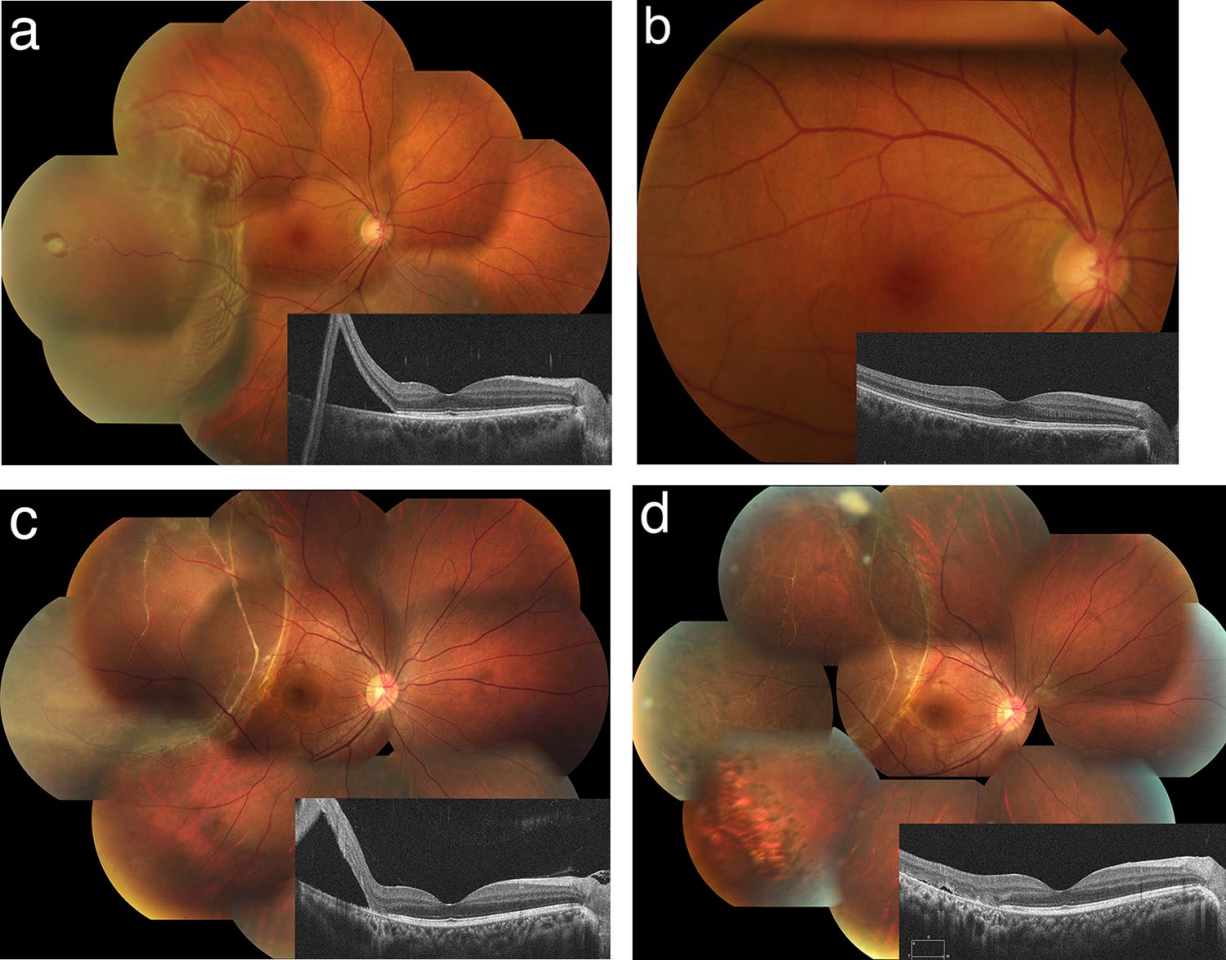
Cases treated with pneumatic retinopexy. **a** The right eye of a 55-year-old female with a history of LASIK surgery. The visual acuity was 1.2 (=20/16). A single retinal break was found at 9 o’clock causing a rhegmatogenous retinal detachment close to the fovea. As this was a typical case meeting the PIVOT criteria, pneumatic retinopexy was chosen for treatment and 100% SF_6_ was injected on the day of presentation. **b** The fundus photo on the next day shows an appropriate volume of gas and the OCT shows complete resolution of the subretinal fluid. Laser retinopexy was performed. **c** The right eye of a 20-year-old moderately myopic female with no ocular symptoms. There was a retinal hole at 10 o’clock in the area of lattice degeneration (not visible on the photo) causative of a long-standing retinal detachment with prominent subretinal proliferation. The location and size of the retinal hole met PIVOT criteria, and the proliferative tissue was not causing visible traction on the retinal hole. **d** After injection of 100% SF_6_, the peripheral retina was reattached allowing photocoagulation to the entire extent of lattice degenerations including the retinal hole. The subretinal fluid gradually decreased until it was completely reabsorbed at 9 months after treatment. *LASIK* laser assisted in situ keratomileusis, *PIVOT* primary rhegmatogenous retinal detachment outcomes randomized trial, *OCT* optical coherence tomography

During the post-PnR period, the PARR following PnR (88.3%) was similar to the results of the PIVOT Trial [4]. The decision to use PnR was influenced by, but not limited to, the PIVOT criteria, with some extension of the criteria as suggested in the literature. These extended indication cases included eyes with a single inferior break (1 case) and with single (4 cases) or multiple breaks (13 cases) in the upper quadrants extending for 2-5 clock hours [19, 28, 34]. When the PARR was calculated independently in eyes that met and extended beyond the PIVOT criteria, the success rate was better in the former but still exceeded 80% in the latter group (90.5% vs. 83.3%), although the difference was not statistically significant (Table 5). These results support the feasibility of PnR both in cases meeting the PIVOT criteria and in cases that may not meet that criteria but are deemed eligible and appropriate based on careful observation and interpretation of the pathology (Fig. 1). PnR in eyes not meeting the PIVOT criteria may be considered controversial, but the relatively favorable PARR of over 80% in the current study suggests its potential use in extended indication cases given the superiority of PnR in terms of reduced invasiveness and better visual recovery demonstrated in multiple studies [4–10].

We considered reattachment after sequential gas injections as a PARR. The fundamental concept and mechanism of PnR is to tamponade the retinal break with the gas bubble and induce chorioretinal adhesion by laser or cryopexy after reattachment around the causative pathologies [1]. However, the size of the bubble injected by a single injection may not be sufficient to reach the goal of the treatment. In such cases, an additional gas injection may be efficacious [28, 31]; reattachment achieved after sequential injections has been considered a favorable outcome as the added step is aligned with the fundamental concept of PnR and is consistent with the definition of primary reattachment in the PIVOT trial [4, 29–31]. In order to interpret the PARR after PnR in this study, it was important to use the same technique, definitions and outcomes as the reference study, since the outcomes of PnR can be impacted remarkably by the experience of the surgeon and the technique adopted for the surgery, as suggested in a meta-analysis [7].

Recently, Pecaku et al. reported a higher PARR in eyes meeting PIVOT criteria with a single break compared to the original cohort in the PIVOT trial [35]. Similarly, the results of this study demonstrate the highest PARR in eyes with a single break meeting the PIVOT criteria (100%) (Table 6). These results strongly support that PARR after PnR is comparable to those after PPV or SB if performed in eyes with the most ideal conditions for PnR. The 71.4% PARR among eyes treated with additional gas injections suggests that a substantial proportion of patients who are heading to failure can be treated successfully with an additional gas injection.

**Table 6:** Primary anatomic reattachment rate in pneumatic retinopexy defined by number of gas injection and number/extent of the retinal break

Number of gas injection and retinal breaks	Primary anatomic reattachment rate	*P*^a^
Number of gas injection		
Single injection (n=46), No. (%)	43/46 (93.5%)	
Meeting PIVOT criteria (n=36), No. (%)	34/36 (94.4%)	0.530
Not meeting PIVOT criteria (n=10)^b^, No. (%)	9/10 (90.0%)	
Additional injection (n=14), No. (%)	10/14 (71.4%)	
Meeting PIVOT criteria (n=6), No. (%)	4/6 (66.6%)	1.000
Not meeting PIVOT criteria (n=8)^c^, No. (%)	6/8 (75.0%)	
Eyes with a single retinal break (n=30)	28/30 (93.3%)	
Meeting PIVOT criteria (n=25)	25/25 (100.0%)	0.023
Not-meeting PIVOT criteria^d^ (n=5)	3/5 (60.0%)	

*PnR* pneumatic retinopexy

^a^Fisher’s exact test; comparison between cases meeting and not-meeting PIVOT criteria

^b^Superior break(s) > 30 degrees in all cases

^c^Superior break(s) > 30 degrees in 7 cases, inferior break in 1 case

^d^A single superior break extending over 30 degrees

Our study did not find superior visual acuity outcomes following PnR compared to PPV (Table 3), a finding that differs from the PIVOT trial results [4]. This could partly be attributed to the retrospective nature of this study and a relatively small sample size. Lens status at follow-up may present another important factor, due to the common practice in Japan of performing PPV combined with cataract extraction and intraocular lens implantation [11, 32]. It is possible that the visual outcomes after PnR were underestimated by lens status while those after PPV were not at all influenced by the presence of cataract since their majority was pseudophakic post-RRD repair [32]. It is important to note that none of the cases treated with PnR in the current study underwent cataract surgery following RRD repair and that there was a lower incidence of cataract formation following PnR compared to PPV reported in the PIVOT trial [4]. This is a significant advantage for PnR in phakic patients as it allows the lens to be preserved with similar visual acuity outcomes compared to patients undergoing PPV with combined cataract surgery.

It is important to note that in this study, the surgeon used social media to remotely learn all steps of PnR as there was no personal access to instructions by an expert. The treating surgeon (KA) joined a social media group to learn PnR and prepared for implementation of PnR for six months by continuously participating in discussions of various cases and asking questions about the principles of the procedure as well as learning from publications and lectures posted within the group [27]. The first case in this study was presented in the group and followed with the advice from an expert (RHM) at each step. As a result, the treating surgeon did not experience any difficulties in performing PnR, and afterwards, all cases were treated without severe complications. This method of learning PnR online contributed to its smooth implementation as has been described in the literature [24–27]. The benefit of online learning in this specific PnR social media group has been reported in a recent survey of the 81 members of the group. The study found a significant increase in the proportion of RRD cases treated with PnR and significant improvements in the success rate of PnR after the surgeons had joined the group [27]. One of the advantages of remote-learning in the group was that the treating method was standardized by the instructing expert; thus, the results presented in the current study can be interpreted without any significant variance from the standardized method utilized in the PIVOT trial.

There are some limitations to this study. The first limitation is the retrospective nature, although variability in case selection and surgical skill were minimized by recruiting patients treated by a single surgeon. Secondly, functional outcomes such as metamorphopsia and aniseikonia were not assessed. These outcomes are important to assess patient vision-related quality of life and should be included in future studies. Another important limitation is the relatively small number of cases included in each group. Since the number of cases in our study was less than the estimated sample size required to detect non-inferiority, comparison between the two groups should be interpreted with caution. It is possible that the comparison of PARR and visual acuity may have reached statistical significance if assessed among larger numbers of cases. On the other hand, consideration of the minimal clinically important difference would also be warranted. However, despite these limitations, we demonstrated the successful implementation of PnR with excellent anatomic and functional outcomes.

To conclude, PnR was performed in more than half of presenting cases during the first few years following PnR implementation at a tertiary care Japanese center. The use of social media to learn this technique highlights the ease of implementation and standardization of the procedures. The excellent anatomic and functional outcomes further support the utility of this minimally invasive procedure as a first-line treatment for RRDs, particularly in those meeting PIVOT trial criteria and those associated with a single retinal break.

## Supplementary Information

Below is the link to the electronic supplementary material.

Supplementary file1 (PDF 39 KB)
