# Supplementary material for: Implementation of pneumatic retinopexy in the Japanese population
Source: Jpn J Ophthalmol. 2025 Jul 25;70(1):166–74. doi: 10.1007/s10384-025-01241-z (PMC12948828; doi:10.1007/s10384-025-01241-z)
Supplement: Supplementary file 1 — Supplementary file1 (PDF 39 KB) [file 10384_2025_1241_MOESM1_ESM.pdf]

Implementation of pneumatic retinopexy in the Japanese population

Supplementary Table Details of failure after pneumatic retinopexy

Case number	1	2	3	4	5	6	7
Age (years)	54	81	56	44	47	60	70
Gender	F	M	M	M	M	M	M
Laterality	R	R	R	L	L	R	L
Refraction (diopters)	-7.75	-3.00	0.00	-4.50	-0.25	-3.50	-3.50
Baseline visual acuity	Hand motion	Hand motion	1.2 (20/16)	1.2 (20/16)	1.2 (20/16)	0.7 (20/28)	1.0 (20/20)
Foveal status	Off	Off	On	On	On	On	Splitting
Lens status	Phakia	IOL	IOL	Phakia	Phakia	Phakia	IOL
Loc of breaks	Sup	Sup	Temp	Sup+inf [a]	Sup	Temp	Sup
Number of breaks	1	3	2	2	1	3	2
Type of breaks	Lattice edge	Hole in lattice	Atrophic hole	Lattice edge	Lattice edge	Perivascular	Flap
Extent of breaks in the detached retina (hours)	1.5	1	1	1	2.5	2	1
Duration>30 days[b]	No	No	Yes	No	No	No	No
Meeting PIVOT criteria	No	Yes	Yes	Yes	No	No	Yes
Reason for failure	Opening of the original break	Unable to keep position (Alzheimer)	Undetected inferior micro hole	Opening of the original break (previously lasered)	Enlargement of the original break (along lattice deg)	Subretinal migration of gas	Opening of the original break (cryopexy)
Number of gas injection	2	2	1	2	2	1	1
Surgical intervention	PPV	PPV	PPV	PPV	PPV	PPV	PPV
Final visual acuity	0.8 (20/25)	0.5 (20/40)	1.2 (20/16)	1.2 (20/16)	1.0 (20/20)	0.8 (20/25)	1.0 (20/20)
Follow-up months	20.3	18.2	13.7	12.5	12.8	9.4	6.1

[a] An Inferior break was located in the attached retina.

[b] Duration between the first onset of visual symptoms and pneumatic retinopexy
